# ASO Authors Reflection: Diagnostic Accuracy of Nipple Discharge Fluid Cytology: A Meta-Analysis and Systematic Review of the Literature

**DOI:** 10.1245/s10434-021-11094-8

**Published:** 2021-11-20

**Authors:** Natasha Jiwa, Daniel Leff

**Affiliations:** 1 Department of Surgery and Cancer, Imperial College London, London, UK; 2 Department of Surgery & Cancer, Imperial College Healthcare NHS Trust, Breast Surgery, London, UK; 3 Hamlyn Centre for Robotic Surgery, Imperial College London, London, UK

## Past

Nipple discharge is the third most frequent complaint of women attending breast clinic. Spontaneous single-duct, blood-stained discharge is widely accepted as a clinical sign warranting further investigation, in the form of imaging, cytology, and often diagnostic surgery.^1^ Nipple smear cytology remains the single most utilised diagnostic modality for investigation of fluid content, although little is known about its overall diagnostic accuracy.

## Present

This study is a meta-analysis of the diagnostic accuracy of nipple discharge fluid cytology for both benign breast disease and breast cancer across three databases, taking into account their quality scoring. The authors concluded that overall, the sensitivity and specificity of nipple discharge cytology was 0.78 (range 0.64–0.93) and 0.43 (range 0.25–0.61) respectively for a benign diagnosis and 0.46 (range 0.30–0.62) and 0.74 (range 0.59–0.88) respectively for breast cancer. Furthermore, patients presenting with blood-stained discharge yielded an overall malignancy rate of 0.58 (range 0.54-0.60) with a positive predictive value (PPV) of 0.27 (95% confidence interval [CI]: 0.17–0.36). Pooled ultrasound sensitivity and specificity was 0.70 (range 0.60–0.80) and 0.58 (95% CI: 0.24–0.91); mammography sensitivity and specificity was 0.38 (95% CI: 0.23–0.52) and 0.79 (95% CI: 0.69–0.90); and MRI sensitivity and specificity was 0.70 (95% CI: 0.61–0.70) and 0.45 (95% CI: 0.20–0.70).^2^ The key implication of this report is that nipple discharge fluid cytology is limited by low sensitivity, which is similar to all stand-alone diagnostic modalities.

## Future

Our recommendation is for patients with single duct nipple discharge a tailored approach to diagnosis is required, given variable accuracy of imaging and cytology in this population. In the conquest to discover superior diagnostic techniques for nipple fluid analysis, emerging technologies must provide diagnostic accuracy, which is greater than cytology, whilst offering advantages in terms of cost, reproducibility, user dependency, and turn-around time.
